# Improved Disease-Free Survival With Adjuvant Chemotherapy After Nephroureterectomy for Upper Tract Urothelial Cancer: Final Results of the POUT Trial

**DOI:** 10.1200/JCO.23.01659

**Published:** 2024-02-13

**Authors:** Alison Jane Birtle, Robert Jones, John Chester, Rebecca Lewis, Katie Biscombe, Mark Johnson, Anthony Blacker, Richard T. Bryan, James W.F. Catto, Ananya Choudhury, Prantik Das, Satinder Jagdev, Thomas Powles, John Wagstaff, Ka Ching Cheung, Fay Cafferty, Emma Hall

**Affiliations:** ^1^ Lancashire Teaching Hospitals NHS Foundation Trust, Preston, United Kingdom; ^2^ University of Manchester, Manchester, United Kingdom; ^3^ University of Central Lancashire, Preston, United Kingdom; ^4^ University of Glasgow, Glasgow, United Kingdom; ^5^ Beatson West of Scotland Cancer Centre, Glasgow, United Kingdom; ^6^ Alder Hey Children's NHS Foundation Trust, Liverpool, United Kingdom; ^7^ The Institute of Cancer Research, London, United Kingdom; ^8^ Newcastle upon Tyne Hospitals NHS Foundation Trust, Newcastle, United Kingdom; ^9^ University Hospitals Coventry and Warwickshire NHS Trust, Coventry, United Kingdom; ^10^ University of Birmingham, Birmingham, United Kingdom; ^11^ University of Sheffield, Sheffield, United Kingdom; ^12^ Sheffield Teaching Hospitals NHS Foundation Trust, Sheffield, United Kingdom; ^13^ University Hospitals of Derby and Burton NHS Foundation Trust, Derby, United Kingdom; ^14^ Leeds Teaching Hospitals NHS Trust, Leeds, United Kingdom; ^15^ Barts Cancer Institute, London, United Kingdom; ^16^ Swansea University, Swansea, United Kingdom

## Abstract

*Clinical trials frequently include multiple end points that mature at different times. The initial report, typically based on the primary end point, may be published when key planned co-primary or secondary analyses are not yet available. Clinical Trial Updates provide an opportunity to disseminate additional results from studies, published in* JCO *or elsewhere, for which the primary end point has already been reported.*

POUT was a phase III, randomized, open-label trial, including 261 patients with muscle-invasive or lymph node–positive, nonmetastatic upper tract urothelial cancer (UTUC) randomly assigned after radical nephroureterectomy to platinum-based chemotherapy (132) or surveillance (129). Primary outcome analysis demonstrated that chemotherapy improved disease-free survival (DFS). At that time, the planned secondary outcome analysis of overall survival (OS) was immature. By February 2022, 50 and 67 DFS events had occurred in the chemotherapy and surveillance groups, respectively, at a median follow-up of 65 months. The 5-year DFS was 62% versus 45%, univariable hazard ratio (HR), 0.55 (95% CI, 0.38 to 0.80, *P* = .001). The restricted mean survival time (RMST) was 18 months longer (95% CI, 6 to 30) in the chemotherapy arm. There were 46 and 60 deaths in the chemotherapy and control arms, respectively. The 5-year OS was 66% versus 57%, with univariable HR, 0.68 (95% CI, 0.46 to 1.00, *P* = .049) and RMST difference 11 months (95% CI, 1 to 21). Treatment effects were consistent across chemotherapy regimens (carboplatin or cisplatin) and disease stage. Toxicities were similar to those previously reported, and there were no clinically relevant differences in quality of life between arms. In summary, although OS was not the primary outcome measure, the updated results add further support for the use of adjuvant chemotherapy in patients with UTUC, suggesting long-term benefits.

## INTRODUCTION

Primary analysis of the POUT trial, demonstrating improved disease-free survival (DFS), supports the use of adjuvant gemcitabine:platinum chemotherapy after nephroureterectomy for patients with muscle invasive upper tract urothelial cancer (renal pelvis or ureter, UTUC).^1^ At the time of initial publication, overall survival (OS) data, a key secondary end point, were immature. We present updated DFS and a prespecified final analysis of OS and other secondary end points.

## PATIENTS AND METHODS

### Study Design

Trial design details have been published previously.^1^ POUT (ClinicalTrials.gov identifier: NCT01993979) was a phase III randomized, open-label trial in which patients with UTUC with muscle-invasive (pT2-T4, Nany) or lymph node–positive (pTany, N1-3), nonmetastatic disease were randomized after radical nephroureterectomy 1:1 to platinum-based adjuvant chemotherapy or surveillance. Chemotherapy was four 21-day cycles of gemcitabine (1,000 mg/m^2^ once per day on days 1 and 8) and either cisplatin (70 mg/m^2^) or, if glomerular filtration rate 30-49 mL/min, carboplatin (AUC 4.5 or 5) once on day 1. The study closed early on advice of the independent data monitoring committee because of superior efficacy in the chemotherapy arm. The trial had ethics approval (11/NW/0782), and participants gave informed consent.

### End Points

The final OS analysis was planned for when ≥88 deaths had been reported or all participants had been followed up for ≥2 years. OS was defined as time from random assignment to death from any cause (censored at date last known to be alive).

We present updated results for the primary end point (DFS) and the secondary end points: metastasis-free survival (MFS), disease-specific survival (DSS), and quality of life (QoL; European Organisation for Research and Treatment of Cancer Quality of Life questionnaire and EQ-5D at 12 and 24 months). In addition, time to second primary tumor in the bladder (TSPB) and late toxicity (6-24 months, Common Terminology Criteria for Adverse Events [CTCAE] v4, with censoring 3 months before recurrence) are reported and we describe subsequent treatments (exploratory end point). TSPB was defined as time from random assignment to the date of diagnosis of second bladder primary (muscle-invasive or non–muscle-invasive), censored at diagnosis of other second primary, date last known to be event-free, or death.

### Statistical Analysis

In addition to methods described previously,^1^ where nonproportional hazards were evident from tests of Schoenfeld residuals,^2^ restricted mean survival time (RMST) was used to estimate differences between arms in average survival time within a 9-year period without assuming a constant hazard ratio.^3^ Analysis was by intention-to-treat with the exception of toxicity (analyzed by treatment received).

## RESULTS

### Participants

Two hundred sixty-one patients (132 chemotherapy; 129 surveillance) were randomly assigned between June 2012 and November 2017 at 57 centers. By February 2022, the median follow-up was 65 months (IQR, 60-84). One participant (chemotherapy arm) withdrew consent for data use and was excluded from analyses. Table 1 shows baseline characteristics.

**TABLE 1.** Participant and Tumor Characteristics at Trial Entry

Characteristic	Surveillance (n = 129)	Chemotherapy (n = 131)	Total (N = 260)
Age, years			
Median	66	69	68
Range	43-88	36-85	36-88
Sex, No. (%)			
Male	83 (64.3)	93 (71.0)	176 (67.7)
Female	46 (35.7)	38 (29.0)	84 (32.3)
Ethnicity, No.			
British	123	118	241
Irish	0	1	1
Indian	2	1	3
Pakistani	1	0	1
Chinese	0	1	1
Other Black background	0	1	1
Other White background	2	5	7
Not specified	1	4	5
Planned chemotherapy regimen,^a^ No. (%)			
Gemcitabine-cisplatin	82 (63.6)	79 (60.3)	161 (61.9)
Gemcitabine-carboplatin	47 (36.4)	52 (39.7)	99 (38.1)
Nodal involvement, No. (%)			
N0	118 (91.5)	118 (90.1)	236 (90.8)
N1+	11 (8.5)	13 (9.9)	24 (9.2)
Microscopic surgical margins, No. (%)			
Positive	14 (10.9)	17 (13.0)	31 (11.9)
Negative	115 (89.2)	114 (87.0)	229 (88.1)
Tumor stage, No. (%)			
T2	30 (23.3)	44 (33.6)	74 (28.5)
T3	88 (68.2)	83 (63.4)	171 (65.8)
T4	11 (8.5)	4 (3.1)	15 (5.8)
Primary tumor location, No.			
Ureter	42	47	89
Renal pelvis	45	47	92
Both	41	37	78
Unknown	1	0	1
No. of lesions, No.			
1	112	109	221
>1	13	18	31
Unknown	4	4	8

Abbreviations: N, node; T, tumor.

^a^ Chemotherapy regimen to be used in the event of random assignment to the chemotherapy arm was specified before random assignment.

### Disease Events

**FIG 1.**
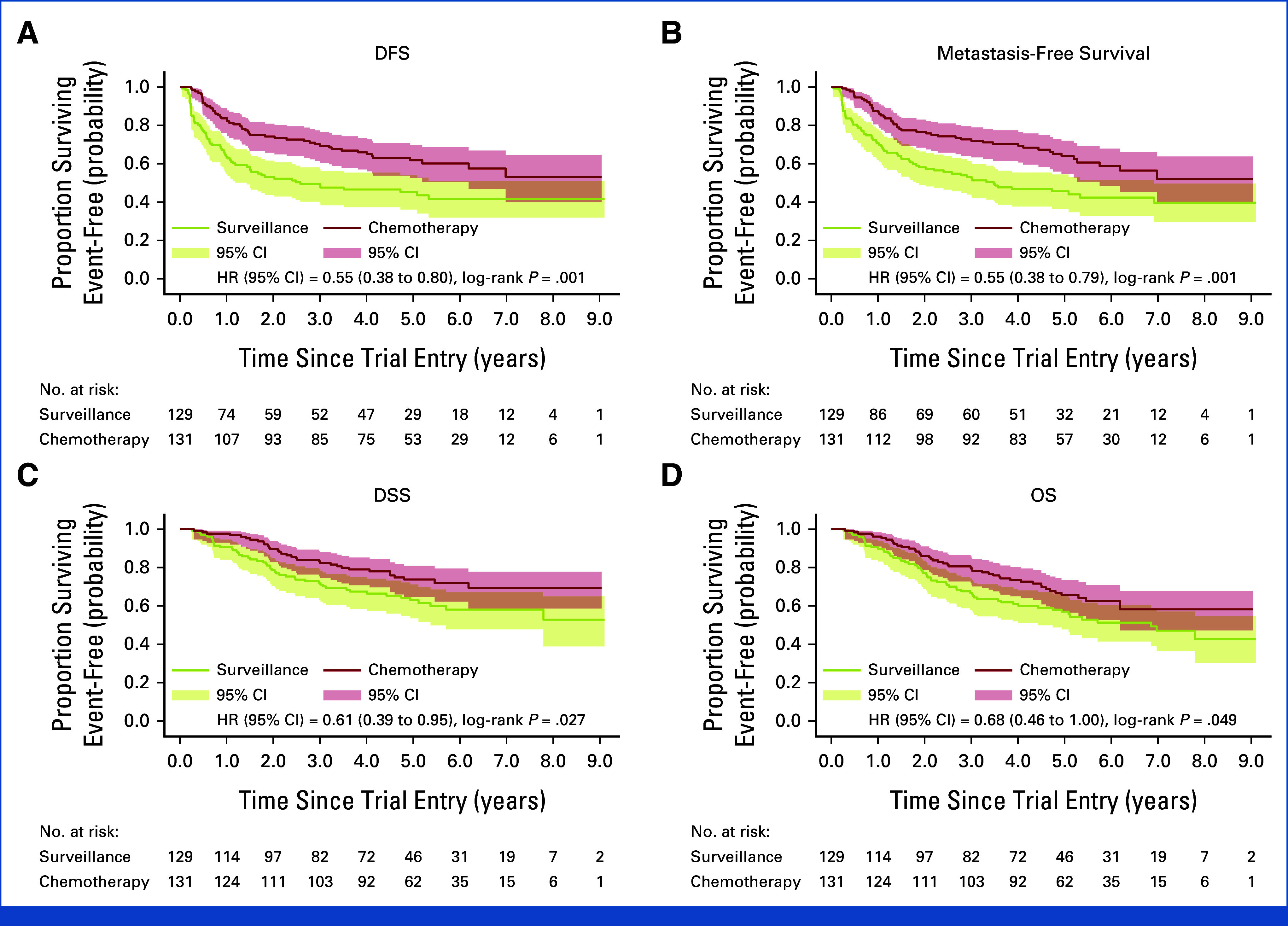
Kaplan-Meier plots with univariable HRs for efficacy analyses (intent-to-treat) showing (A) DFS, (B) metastasis-free survival, (C) DSS, and (D) OS. DFS, disease-free survival; DSS, disease-specific survival; HR, hazard ratio; OS, overall survival.

There were 50 and 67 DFS events in the chemotherapy and surveillance groups, respectively. Risk of recurrence or death was reduced in patients allocated to chemotherapy (5-year DFS 62% *v* 45%; univariable hazard ratio [HR], 0.55 [95% CI, 0.38 to 0.80], *P* = .001; multivariable HR, 0.58 [95% CI, 0.40 to 0.84], *P* = .004, adjusted for nodal status, planned chemotherapy regimen, margin status, and pathologic stage; Fig 1A).

**FIG 2.**
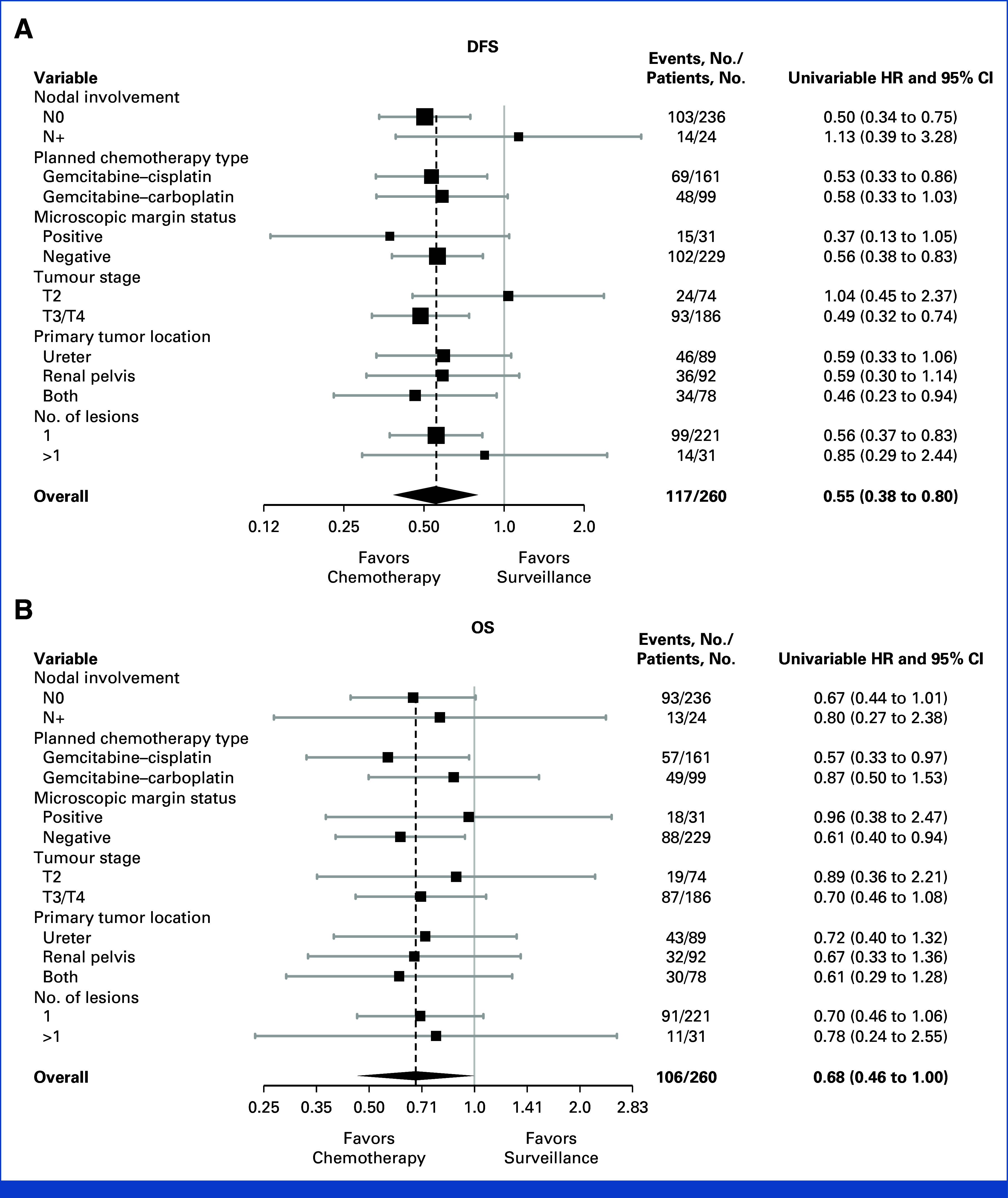
Forest plots showing treatment effects according to key baseline factors and planned chemotherapy regimen for (A) DFS and (B) OS. DFS, disease-free survival; HR, hazard ratio; OS, overall survival.

Nonproportional hazards were evident, and the RMST for DFS was 72 and 54 months, respectively, an 18-month improvement in the chemotherapy arm (95% CI, 6 to 30, *P* = .003). The treatment effect was consistent across subgroups (Fig 2A). MFS and DSS results similarly suggested a benefit of chemotherapy in Cox models (Figs 1B and 1C) and in RMST for MFS where nonproportional hazards were evident (18-month improvement, 95% CI, 6 to 29, *P* = .002).

**FIG A1.**
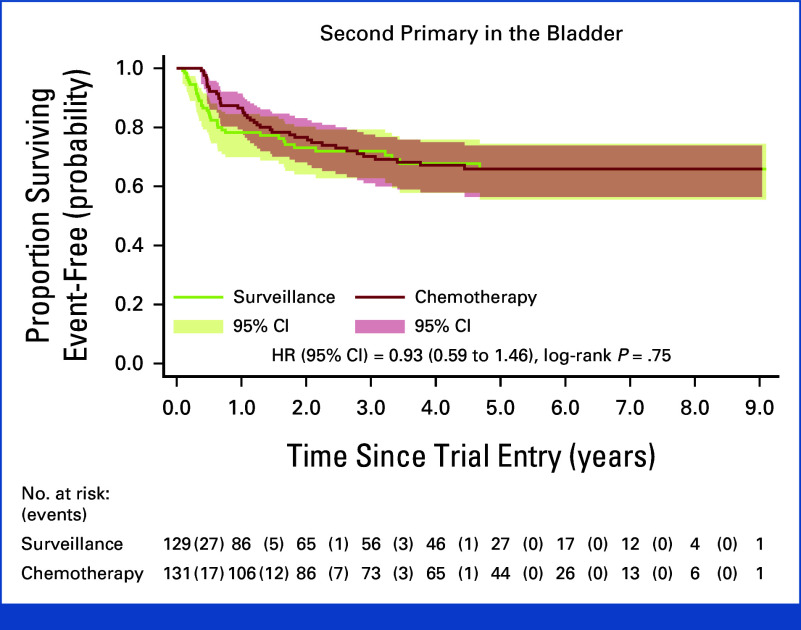
Kaplan-Meier plot with univariable HR for time to second primary cancer in the bladder (intent-to-treat analysis). HR, hazard ratio.

There was no impact of chemotherapy on TSPB (40 events in 131 patients *v* 37 events in 129 patients in the surveillance arm; Appendix Fig A1, online only).

Systemic treatment for recurrence was more common in the surveillance arm (45 of 71 patients with recurrence, 63% *v* 23 of 47, 49%; Appendix Table A1).

### OS

There were 46 and 60 deaths in the chemotherapy and surveillance groups, respectively; 33 of 46 (72%) and 48 of 60 (80%) were due to urothelial cancer (Appendix Table A2). There was a trend toward improved survival in patients allocated to chemotherapy (5-year OS 66% *v* 57%; univariable HR, 0.68 [95% CI, 0.46 to 1.00], *P* = .049; multivariable HR, 0.76 [95% CI, 0.51 to 1.12], *P* = .17) (Fig 1D). The RMST was 78 and 67 months, an 11-month OS improvement with chemotherapy (95% CI, 1 to 21, *P* = .036). The treatment effect was consistent across subgroups (Fig 2B).

### Adverse Events and QoL

CTCAE grade ≥3 rates between 6 and 24 months were similar in both groups (40 of 240, 16.7%, Appendix Table A3). No important differences in QoL were observed (Appendix Table A4).

## DISCUSSION

Primary results from POUT have already changed practice on the basis of the DFS benefit.^4^ The validity of changing practice on the basis of DFS alone has been reaffirmed by recent regulatory approvals for adjuvant nivolumab in invasive urothelial cancer, including UTUC.^4^ Although preventing relapse is of likely clinical benefit in its own right, one key purpose of adjuvant therapy is to delay or prevent cancer death. Because of the rarity of UTUC, it was impractical to conduct a trial with OS as a primary end point. Furthermore, since POUT was stopped early on the basis of superior DFS with chemotherapy, power for OS analysis was reduced. Nevertheless, a statistically significant OS advantage was seen in univariable analysis (*P* = .049) and, although nonsignificant, multivariable modeling showed a consistent positive trend. The presence of nonproportional hazards may also affect the power of these analyses^3^; RMST results, which account for this, show a statistically significant OS benefit of 11 months over a 9-year period, with the peak benefit between 3 and 4 years. Combined with improvements in MFS and DSS, these results add weight to the sustained DFS benefit confirmed here.

Although carboplatin is considered by many to be less effective than cisplatin in urothelial cancer,^5^ nephroureterectomy (by its nature) results in reduced renal function. Hence, it was important, for generalizability of results, to include a safe option for delivering platinum-based chemotherapy for those with impaired renal function. Subgroup results from the POUT primary analysis left some uncertainty about the value of carboplatin for those patients.^1^ Although not powered for a formal test of interaction, updated HRs (Fig 2) suggest a consistent benefit of chemotherapy, regardless of the regimen, supporting inclusion of these patients in the treatment recommendation. Other recent data also suggest that the utility of carboplatin compared with cisplatin in urothelial cancer has been underestimated.^6,7^

The POUT primary analysis showed acceptable levels of acute toxicity with chemotherapy, in line with previous reports.^8^ In the current analysis, data on both clinician-reported toxicity and patient-reported QoL provide reassurance that there are no important long-term adverse impacts, which might offset the benefits. Systemic therapy on relapse was less frequent in those who received adjuvant chemotherapy than those in the surveillance group. This may reflect the lack of effective, approved second-line therapies in the UK during most of the POUT follow-up period. By contrast, control arm patients could access frontline platinum-based chemotherapy on relapse. We speculate that this difference between arms is unlikely to have had any significant impact on the trial end points.

While chemotherapy reduces time to metastasis, it appeared to have no impact on the evolution of second primary formation in the bladder. The extent to which such tumors are clonally related to UTUC has varied in previous studies.^9-12^ The pattern here may suggest that, particularly, later-forming tumors could be the result of a new, in situ oncogenic process; notwithstanding, such temporal relationships remain to be fully elucidated.

In summary, updated outcomes from the POUT trial add further support to the value of adjuvant systemic gemcitabine:platinum combination chemotherapy after nephroureterectomy for UTUC.

## PRIOR PRESENTATION

Presented in part at the 2021 ASCO Genitourinary Cancer Symposium, virtual, February 11-13, 2021.

## SUPPORT

Supported by Cancer Research UK (C8262/A13324, CRUK/11/027, and program grants which support the work of Institute of Cancer Research-Clinical Trials and Statistics Unit C1491/A15955, C1491/A25351).

## CLINICAL TRIAL INFORMATION

NCT01993979 (POUT)

## DATA SHARING STATEMENT

Deidentified individual participant data, together with a data dictionary defining each field in the set, will be made available to other researchers on request. Trial documentation including the protocol is available online. The ICR-CTSU supports wider dissemination of information from the research it conducts and increased cooperation between investigators. Trial data are obtained, managed, stored, shared, and archived according to ICR-CTSU standard operating procedures to ensure the enduring quality, integrity, and utility of the data. Formal requests for data sharing are considered in line with ICR-CTSU procedures, with due regard given to funder and sponsor guidelines. Requests are via a standard proforma describing the nature of the proposed research and extent of data requirements. Data recipients are required to enter a formal data sharing agreement, which describes the conditions for release and requirements for data transfer, storage, archiving, publication, and intellectual property. Requests are reviewed by the trial management group in terms of scientific merit and ethical considerations, including patients' consent. Data sharing is undertaken if proposed projects have a sound scientific or patients' benefit rationale, as agreed by the trial management group and approved by the independent data monitoring and steering committee, as required. Restrictions related to patients' confidentiality and consent will be limited by aggregating and anonymizing identifiable patients' data. Additionally, all indirect identifiers that could lead to deductive disclosures will be removed in line with ICR-CTSU data sharing guidelines.

## APPENDIX

**TABLE A1.** Details of Treatment for Recurrence

Recurrence Treatment	Surveillance, No. (%)^a^	Chemotherapy, No. (%)^a^	Total, No. (%)^a^
Patients with a recurrence	71	47	118
Systemic therapies	45 (63.4)	23 (48.9)	68 (57.6)
Platinum chemotherapy^b^	39 (54.9)	13 (27.7)	52 (44.1)
Nonplatinum chemotherapy^b^	1 (1.4)	3 (6.4)	4 (3.4)
Immunotherapy^b^	8 (11.3)	6 (12.8)	14 (11.9)

^a^ Percentage of patients with a recurrence treated in this way (ie, denominator is the number of patients who experienced a recurrence of any kind).

^b^ Categories are not mutually exclusive since patients might have received multiple treatments.

**TABLE A2.** Causes of Death

Cause of Death	Surveillance (n = 60), No. (%)	Chemotherapy (n = 46), No. (%)	Total (n = 106), No. (%)
UTUC	48 (80.0)	33 (71.7)	81 (76.4)
Bladder cancer	5 (8.3)	5 (10.9)	10 (9.4)
Other malignancies	2^a^ (3.3)	1^b^ (2.2)	3 (2.8)
Myocardial infarction	1 (1.7)	0 (0.0)	1 (0.9)
Respiratory causes	1 (1.7)	0 (0.0)	1 (0.9)
Cardiovascular issues	1 (1.7)	1 (2.2)	2 (1.9)
Infection	1 (1.7)	4 (8.7)	5 (4.7)
Other	0 (0.0)	1^c^ (2.2)	1 (0.9)
Not specified	1 (1.7)	1 (2.2)	2 (1.9)

Abbreviation: UTUC, upper tract urothelial cancer.

^a^ Small-cell carcinoma of left lung (n = 1); colorectal (n = 1).

^b^ AML.

^c^ Gastric bleed (n = 1).

**TABLE A3.** Late Toxicity Reported Between 6 and 24 Months Postrandomization (censored within 3 months of progression)

Follow-Up Time (postrandomization)	Maximum CTCAE Grade Reported	Surveillance, No. (%)	Chemotherapy, No. (%)	Total, No. (%)
Month 6 (n = 240) Surveillance (n = 117) Chemotherapy (n = 123)	0	46 (39.3)	42 (34.1)	88 (36.7)
	1	41 (35.0)	46 (37.4)	87 (36.3)
	2	12 (10.3)	24 (19.5)	36 (15.0)
	3	13 (11.1)	7 (5.7)	20 (8.3)
	4	1 (0.9)	1 (0.8)	2 (0.8)
	Missing	2 (1.7)	3 (2.4)	5 (2.1)
	Grade <3	101 (86.3)	112 (91.1)	213 (88.8)
	Grade 3-4	14 (12.0)	8 (6.5)	22 (9.2)
	Missing	2 (1.7)	3 (2.4)	5 (2.1)
Month 12 (n = 222) Surveillance (n = 103) Chemotherapy (n = 119)	0	39 (37.9)	48 (40.3)	87 (39.2)
	1	36 (35.0)	43 (36.1)	79 (35.6)
	2	15 (14.6)	16 (13.4)	31 (14.0)
	3	7 (6.8)	8 (6.7)	14 (6.3)
	4	0 (0.0)	1 (0.8)	1 (0.5)
	5	0 (0.0)	1 (0.8)	1 (0.5)
	Missing	6 (5.8)	2 (1.7)	8 (3.6)
	Grade <3	90 (87.4)	107 (89.9)	197 (88.7)
	Grade 3-5	7 (6.8)	10 (8.4)	17 (7.7)
	Missing	6 (5.8)	2 (1.7)	8 (3.6)
Month 18 (n = 198) Surveillance (n = 91) Chemotherapy (n = 107)	0	43 (47.3)	41 (38.3)	84 (42.4)
	1	19 (20.9)	41 (38.3)	60 (30.3)
	2	17 (18.7)	15 (14.0)	32 (16.2)
	3	6 (6.6)	8 (7.5)	14 (7.1)
	4	0 (0.0)	1 (0.9)	1 (0.5)
	Missing	6 (6.6)	2 (1.9)	8 (4.0)
	Grade <3	79 (86.8)	97 (90.7)	176 (88.9)
	Grade 3-4	6 (6.6)	9 (8.4)	15 (7.6)
	Missing	6 (6.6)	1 (0.9)	7 (3.5)
Month 24 (n = 177) Surveillance (n = 83) Chemotherapy (n = 94)	0	35 (42.2)	36 (38.3)	71 (40.1)
	1	27 (32.5)	31 (33.0)	58 (32.8)
	2	15 (18.1)	18 (19.1)	33 (18.6)
	3	6 (7.2)	7 (7.4)	13 (7.3)
	4	0 (0.0)	2 (2.1)	2 (1.1)
	Grade <3	77 (92.8)	85 (90.4)	162 (91.5)
	Grade 3-4	6 (7.2)	9 (9.6)	15 (8.5)
Maximum overall (n = 240) Surveillance (n = 117) Chemotherapy (n = 123)	0	22 (18.8)	9 (7.3)	31 (12.9)
	1	38 (32.5)	57 (46.3)	95 (39.6)
	2	33 (28.2)	32 (26.0)	65 (27.1)
	3	21 (17.9)	21 (17.1)	42 (17.5)
	4	1 (0.9)	3 (2.4)	4 (1.7)
	5^a^	0 (0.0)	1 (0.8)	1 (0.4)
	Grade <3	95 (81.2)	98 (79.7)	193 (80.4)
	Grade 3-5	22 (18.8)	25 (20.3)	47 (19.6)

Abbreviation: CTCAE, Common Terminology Criteria for Adverse Events.

^a^ One grade 5: death because of gastric bleeding.

**TABLE A4.** Differences Between Treatment Groups in Mean Functional and Symptomatic Quality-of-Life Scales (EORTC-QLQ-C30) Reported at 12 and 24 Months Postrandomization

Quality-of-Life Scale Type	Item	12 Months^a^			24 Months^b^		
		Difference^c^	99% CI^d^	*P* ^d^	Difference^c^	99% CI^d^	*P* ^d^
Functional scales (high scores indicate healthy functioning)	Global health status/QoL	3.99	–4.53 to 12.50	.22	4.90	–5.14 to 14.94	.20
	Health state today (EQ-5D)	4.42	–3.93 to 12.78	.17	–2.41	–11.77 to 6.94	.50
	Physical functioning	–4.17	–11.35 to 3.01	.13	–0.60	–8.25 to 7.06	.84
	Role functioning	–2.27	–13.56 to 9.03	.60	0.26	–11.73 to 12.25	.95
	Emotional functioning	4.42	–3.22 to 12.06	.13	5.64	–3.24 to 14.53	.10
	Cognitive functioning	–0.81	–8.39 to 6.76	.78	0.49	–7.72 to 8.69	.88
	Social functioning	1.43	–10.42 to 13.29	.75	0.89	–9.64 to 11.42	.83
Symptomatic scales (high scores indicate a high level of symptoms)	Fatigue	–2.71	–11.78 to 6.36	.44	–7.26	–16.46 to 1.93	.04
	Nausea and vomiting	–1.97	–7.61 to 3.66	.36	0.56	–6.87 to 7.98	.85
	Pain	–2.10	–12.47 to 8.27	.60	0.64	–10.78 to 12.05	.88
	Dyspnea	3.89	–5.68 to 13.46	.29	6.13	–4.95 to 17.21	.15
	Insomnia	–4.56	–15.65 to 6.52	.28	–8.98	–20.65 to 2.69	.05
	Appetite loss	–3.48	–12.65 to 5.69	.32	–4.74	–13.79 to 4.32	.17
	Constipation	–4.41	–15.29 to 6.47	.29	1.20	–9.15 to 11.54	.76
	Diarrhea	–1.15	–7.93 to 5.63	.66	1.61	–5.30 to 8.53	.54
	Financial difficulties	–5.71	–14.19 to 2.76	.08	–1.06	–9.23 to 7.12	.74

Abbreviation: EORTC-QLQ-C30, European Organisation for Research and Treatment of Cancer Quality of Life questionnaire.

^a^ Surveillance (n = 72), chemotherapy (n = 83).

^b^ Surveillance (n = 59), chemotherapy (n = 73).

^c^ Differences in mean scores between the trial arms (chemotherapy – surveillance); a difference of >10 points would be considered clinically important, with positive differences indicating an improvement with chemotherapy for functional scales and a detrimental effect of chemotherapy for a symptomatic scale.

^d^ 99% CIs and *P* values from analysis of covariance models adjusting for baseline score on the same subscale; *P* < .01 were considered statistically significant to allow for multiple testing.
